# Determinants of Cocoa Farmers’ Compliance with Agrochemical Safety Precautions in Ogun and Osun States, Nigeria

**DOI:** 10.3390/toxics10080454

**Published:** 2022-08-06

**Authors:** Abayomi Samuel Oyekale

**Affiliations:** Department of Agricultural Economics and Extension, North-West University Mafikeng Campus, Mmabatho 2735, South Africa; abayomi.oyekale@nwu.ac.za

**Keywords:** cocoa, safety compliance indicators, health risks, agrochemicals, personal protective equipment

## Abstract

Cocoa is one of the major cash crops in Nigeria and its production is directly related to the effective utilization of agrochemicals. This paper analysed the factors influencing cocoa farmers’ compliance with agrochemical safety regulations. The data were collected from 326 cocoa farmers from Ogun and Osun states, using multi-stage sampling procedures. The data were analysed with Principal Component Analysis (PCA) and an Endogenous Tobit Regression model. The results showed that majority of the farmers were primarily growing cocoa and average ownership of personal protective equipment (PPE) was less than two. Awareness of manufacturers’ instructions was high for insecticides and fungicides, while majority of the farmers were not eating or drinking while handling agrochemicals. Safety compliance indicators were significantly influenced (*p* < 0.10) by farmers’ ownership of PPE, education, age, awareness of manufacturers’ safety instructions and health status. It was concluded that utilization of PPE was very low among the farmers and efforts to facilitate safety training on the use of different agrochemicals will facilitate safety compliance through proper understanding of manufacturers’ instructions.

## 1. Introduction

Cocoa remains one of the foremost crops in some African countries, contributing significantly to rural employment, foreign exchange earnings and agriculture’s Gross Domestic Product (GDP). The importance of cocoa to the rural economy in the producing countries can be best understood from the fact that about 300,000 farmers in Nigeria, 600,000 farmers in Cameroon [1], more than 800,000 farmers in Ghana [2,3,4] and more than 1 million farmers in Cote D’Ivoire [3] are primarily engaged in cocoa cultivation. After a drastic decline in Nigeria’s cocoa production due to the oil boom in the 1970s, cocoa rebirth initiative was implemented in 1999, at which time total production stood at 170,000 tons [5]. Although Nigeria’s cocoa output has recently increased, with available statistics putting production at 340,163 tons [1], Nigeria is yet to regain her lost global position in cocoa production [5]. Currently, Nigeria ranks fourth in global cocoa output, after Cote D’Ivoire with 1,2000,000 tons, Ghana with 800,000 tons and Indonesia with 739,483 tons [1]. 

The Cocoa Farmers’ Association of Nigeria (CFAN) recently pledged to increase cocoa output to 500,000 tons by 2024 [6]. This is a commendable way to diversify Nigeria’s sources of income and stimulate sustainable economic recovery among cocoa farmers, as the country recovers from the economic downturns of the COVID-19 pandemic. However, the optimization of cocoa productivity depends on many factors, among which adequate agronomic practices and favourable weather conditions are notable. Specifically, cocoa performs optimally under a relative humidity of not more than 65%, rainfall of between 1000 mm and 3000 mm, altitude of less than 400m above seas level and adequate temperature of between 18 °C and 32 °C [7,8,9]. However, these weather conditions also promote the development of cocoa pests and pathogens that cause diseases, such as black pod, frosty pod rot, witches’ broom, cocoa swollen shoot and vascular streak dieback [10,11]. These pests and diseases are controlled with the use of agrochemicals [12], which often compromise the health of many farmers [13,14] due to less compliance with recommended safety practices. Consumers also worry about the health implications of agrochemical usage due to perpetual contamination of cocoa beans and surface/ground water channels [15,16,17]. Despite the regulatory role being played by some international organizations to ensure safety of farm workers in the use of agrochemicals [18,19,20,21], mishandling and misuse of agrochemicals are now sources of global public-health concern. Specifically, it has been noted that, globally, about 740,000 annual cases of unintentional acute pesticide poisoning (UAPP) exist, with 7446 fatal cases [22].

Moreover, cocoa farmers are often exposed to some adverse health impacts of agrochemical exposure due to unregulated infiltration of banned pesticides in some cocoa producing countries and low compliance with essential safety and protective practices. More importantly, many African cocoa farmers do not follow prescribed safety precautions [23,24,25]. This is subjecting them to serious occupational health hazards, with some recorded cases of death and permanent disability resulting from misapplication of agrochemicals [17,26]. Some previous studies have explored the determinants of safety perception in agrochemical handling and compliance among cocoa farmers. This is because understanding the correlates of farmers’ compliance with safety precautions in handling agrochemicals is of paramount relevance to the effective design of rural public health interventions, environmental safety and cocoa productivity.

In a study that was conducted among Ghanaian cocoa farmers, it was found that the majority of the farmers did not own agrochemical safety kits, although the number of PPE owned significantly decreased the incidence of skin infections after applying agrochemicals [27]. In another study in Ghana, Miyittah et al. [28] found that the use of PPE was enhanced by access to advice from agricultural extension officers and agrochemical retail outlets, while it declined among female cocoa farmers and residents of Suaman district. In Nigeria, Oludoye et al. [29] reported on the inadequacy of agrochemical safety information that cocoa farmers receive from agrochemical retail outlets, while other stakeholders in the production and marketing chains were not helping with any safety information. Another study among cocoa farmers in Cameroon found that domestic reuse of agrochemical containers significantly decreased among the farmers that engaged in agriculture as the primary occupation, formally educated and residents in the south-west region, while being male increased it. It was further found that the number of PPE worn during agrochemical application significantly increased among farmers in the south-west region, having good health and safety awareness, but decreased with indicators of agrochemical contacts [30].

Olutegbe and Sanni [31] found that, among Nigerian cocoa farmers, the good agricultural practices (GAP) that cocoa farmers were least compliant with were of high environmental, economic and health consequences. In addition, the level of knowledge, economic returns and perception of health status were associated with GAP compliance. In a study by Agbongiarhuoyi and Fawole [32], Nigerian cocoa farmers’ compliance with standard pesticide safety practices decreased with age and access to approved pesticides, while it increased with income and access to information. Tijani [33] found that many cocoa farmers in Ondo state were not complying with necessary precautions for the safe handling of agrochemicals, with several health consequences. Osei-Owusu and Owusu-Achiaw [12] found low use of PPE among cocoa farmers in Ghana, with the lowest used being goggles, gloves and masks at 5%, 10.77% and 31%, respectively. In addition, in Ghana, while many cocoa farmers were using disapproved agrochemicals [12,34], the factors that hinder effective use of PPE were high cost, discomfort and non-availability [12].

There is a need to increase cocoa productivity as part of the post-COVID economic recovery strategy in Nigeria, without compromising the health of farmers. The objective of this paper is to analyse the factors influencing cocoa farmers’ compliance with agrochemical safety regulations. The study is contributing to the existing body of knowledge by including four different forms of agrochemicals that are utilized for cocoa agriculture. It is hypothesized that cocoa farmers’ demographic characteristics and other related variables do not significantly influence compliance with agrochemicals’ safety precautions. 

## 2. Materials and Methods

### 2.1. Data and Sampling Procedures

The data for this study were collected from cocoa farmers in Ogun and Osun states by trained enumerators. The farmers were selected using multi-stage sampling procedures. Stage one involves a purposive selection of Osun and Ogun states among the major cocoa-growing states in the south-west zone. These states were selected because of their relative proximity to each other and the need to understand cocoa farmers’ safety behaviours in agrochemical utilization in coca-producing areas that have been under-represented in many previous studies [35]. More importantly, the proximity of Ogun state to Benin republic and Lagos underscores a very high possibility of easy inflow of banned agrochemicals, thereby necessitating serious compliance with standard safety precautions [36]. At the second stage, two cocoa-growing local government areas were randomly selected from each of the selected states. The third stage involves listing of cocoa-growing villages within each of the selected local government areas. This exercise was facilitated by contacting farmers and agricultural extension officers. 

Based on estimated cocoa farmers in the selected local government areas within the two states, the sample size ($n^{\prime }$) was calculated using an online sample size calculator as [37]:

$$n^{\prime }=\frac{n}{\left(1+\frac{z^{2}*\hat{p}\left(1-\hat{p}\right)}{\varepsilon^{2}N}\right)}$$

where $\frac{n=z^{2}*\hat{p}(1-\hat{p})}{\varepsilon^{2}}$, *z* is the *z*-score at 95 confidence level (1.96), $\hat{p}$ is the population proportion (0.5), ε is margin of error (0.05) and N is the estimated population size (7500). Based on the above, a sample size of 366 was estimated. Although the study aimed to interview 335 cocoa farmers, due to limitations in research budgets and other logistics issues, only 326 were successfully interviewed. This implies a 97.51% response rate. Sample sizes were allocated to each local government area based on estimated number of cocoa farmers. Within the local government areas, samples were also allocated based on the estimated population of cocoa farmers in the selected villages. Cocoa farmers were thereafter randomly selected and interviewed in their houses.

The questionnaire comprises three sections which are socioeconomic characteristics of the households’ heads, agrochemical safeguard measures and occupational health hazards. The questionnaire was designed in the English language. However, translation was made into Yoruba language by the enumerators if the farmers did not understand English. The consent of farmers was sought before interview and their participation in the survey was voluntary. This implies that they could withdraw from participating at any point of the interviews. Table 1 shows the distribution of the respondents across the selected villages.

### 2.2. Limitations of the Study

This study is taken as a pilot study and just like any other research, it faced some factors that limited its scope. The major constraint was lack of sufficient money to cover wider cocoa-production areas in the south-west geopolitical zone. Specifically, four out of several LGAs where cocoa is produced were covered and efforts ensured adequate sampling to enhance representativeness. The study also relied on farmers’ reported compliance which could not be personally verified because of time and other logistic constraints. Future studies with sufficient funding can explore the possibility of observing farmers while applying agrochemicals and inspection of the different brands of agrochemical being used to detect their compliance with some national regulations. 

### 2.3. Analytical Methods

#### 2.3.1. Construction of Safety Compliance Indicators

There are different dimensions of compliance with safety procedures in the application of agrochemicals by cocoa farmers. This study used Principal Component Analysis (PCA) to compute indicators of safety using their responses to four safety-related questions. The first question is on whether a farmer follows the recommended instructions by the manufacturers (Yes = 1, 0 otherwise). The second is on if the farmers spray agrochemicals along the wind direction (Yes = 1, 0 otherwise). The third is on compliance with the instruction not to eat or drink while applying agrochemicals (Yes = 1, 0 otherwise). The fourth question is on whether a farmer always wears each of the recommended kit items (hand gloves, safety boots, goggles, protective clothes and ventilation mask) while spraying agrochemicals (Yes = 1, 0 otherwise). Indicators of compliance were separately generated for each of the agrochemicals—insecticides, herbicides, fertilizers and fungicides.

#### 2.3.2. Endogenous Regressor Tobit Model

The determinants of safety compliance indicators were analysed with Tobit regression model. However, it was noted that the number of safety kits that a farmer owns is a potential determinant and the understanding of its potential endogeneity compels the use of Endogenous Regression Tobit Model. This model is appropriate to safeguard the estimated parameters from conventional Tobit regression from suffering inconsistency and biasness.

(1) $$Y_{1i}^{*}=X_{1i}\gamma +Y_{2i}\beta +u_{i}$$

(2) $$Y_{2i}=X_{1i}\pi_{1}+X_{2i}\pi_{2}+v_{i}$$

where *i* denotes the number of cocoa farmers (1, …, n), $Y_{2i}$ is the endogenous regressors, $X_{1i}$ is a $1xm_{1}$ vector of exogenous variables and $X_{2i}$ is $1xm_{2}$ vector of the instrumental variables. In Equation (1), γ and β are the structural parameters while the reduced-form parameters ($\pi_{1}$ and $\pi_{2}$) are presented in Equation (2). It should be noted that the structural parameters of the model in Equation (1) can only be estimated if $m_{2}=k$ (exactly identified) or $m_{2}>k$ (over identified). In Equation (1), the dependent variable—safety compliance indicator—was computed with PCA using STATA 17 software. The exogenous regressors are: married (yes = 1, 0 otherwise), male (yes = 1, 0 otherwise), illiterate (yes = 1, 0 otherwise), primary occupation farming (yes = 1, 0 otherwise), household size, years of cocoa farming, aware of insecticide application safety measures (yes = 1, 0 otherwise), aware of herbicide application safety measures (yes = 1, 0 otherwise), aware of fertilizer application safety measures (yes = 1, 0 otherwise), aware of fungicide application safety measures (yes = 1, 0 otherwise) and age of farmers (years). The suspected endogenous regressor is the number of safety kits that the farmers owned while the instrumental variables are cocoa land area (hectares), local government area dummy variables (Yewa South—yes = 1, 0 otherwise; Ayedaade—yes = 1, 0 otherwise; and Irewole—yes = 1, 0 otherwise) and health status dummy variables (Very good—yes = 1, 0 otherwise; Good—yes = 1, 0 otherwise; Fair—yes = 1, 0 otherwise; and Poor—yes = 1, 0 otherwise). Selection of adequate instruments requires that no significant association exists between the dependent variable and the instruments, while there must be significant association between instruments and endogenous regressor. The models in Equations (1) and (2) were estimated with *ivtobit* command of STATA 17 [38].

## 3. Results

### 3.1. Cocoa Farmers’ Demographic Characteristics

Table 2 shows the results of the distribution of cocoa farmers’ demographic characteristics across the selected LGAs. It reveals that the majority of the cocoa farmers were generally males across the LGAs. Specifically, 91.7% of all farmers were male. Further, 27.6% of all the farmers had no formal education, with Yewa South LGA having the highest proportion (40.9%). Cocoa farming was a primary occupation for 89.6% of the respondents. The average household size across all the LGAs was about seven members. Average age of the farmers was above 50 in all the LGAs, while most of the farmers had above 20 years of cocoa farming experience. The average area of cocoa farms was 7.714 hectares, with respondents from Ayedaade and Irewole LGAs having an average of 9 hectares. The average number of personal protective equipment (PPE) that was owned by the farmers was 1.574. The majority of the farmers ranked their health status as excellent or very good. Specifically, 26.4% and 45.1% of the farmers indicated their health status was excellent and very good, respectively.

**Figure 1:**
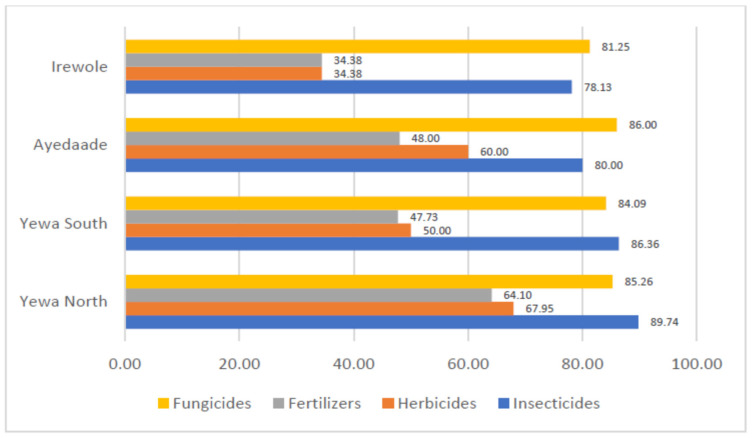
Awareness of manufacturers’ instructions in handling agrochemicals by cocoa farmers.

Figure 1 shows the awareness levels of agrochemical manufacturers’ instructions by cocoa farmers. It reveals that fungicides and insecticides had the highest awareness levels across the selected LGAs. Specifically, 86.00% and 85.26 % of the farmers from Ayedaade and Yewa North LGAs, respectively, indicated awareness of manufacturers’ instructions on the use fungicides. With 89.74% and 86.36% awareness levels, cocoa farmers from Yewa North and Yewa South, respectively, had the highest percentage for insecticides. Awareness of manufacturers’ instructions was generally low for fertilizers, with only 34.38% of the farmers indicating awareness in Irewole LGA.

**Figure 2:**
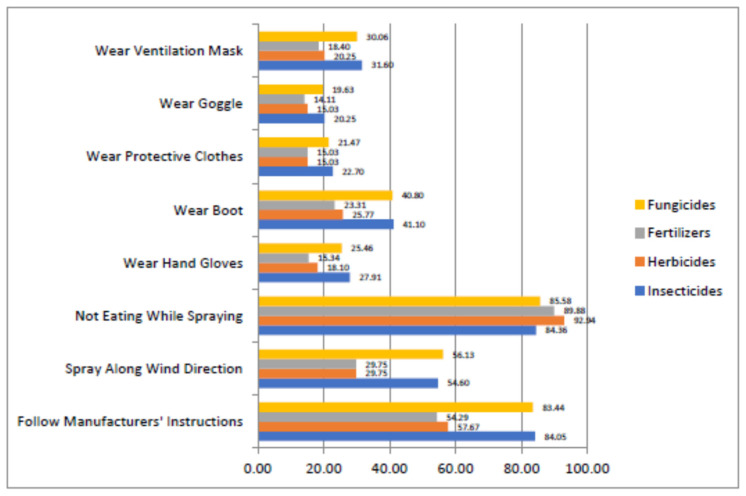
Cocoa farmers’ compliance with selected safety procedures when using agrochemicals.

Figure 2 shows that the majority of the farmers would not eat or drink when applying agrochemicals. Specifically, 92.94% of the farmers would not eat or drink when handling herbicides and 89.88% would comply when applying fertilizers. The manufacturers’ instructions are followed by 84.05% and 83.44% of the farmers when applying insecticides and fungicides, respectively. However, while 56.13% and 54.60% of the cocoa farmers would, respectively, spray fungicides and insecticides along the wind direction, only 29.75% would follow this guideline when applying herbicides and fertilizers. The figure further shows that PPE was generally not used by the majority of farmers. More specifically, wearing safety boots records the highest percentages among the farmers, with 41.10% and 40.80% for application of insecticides and fungicides, respectively. In addition, 31.60% and 30.06% of the farmers wear ventilation masks when applying insecticides and fungicides, respectively. Protective clothes are worn by 21.47% of the farmers when using fungicides, while only 15.03% wear them when handling herbicides and fertilizers. Goggles are worn by only 20.25% and 21.47% of the farmers when applying insecticides and fungicides, respectively. 

**Figure 3:**
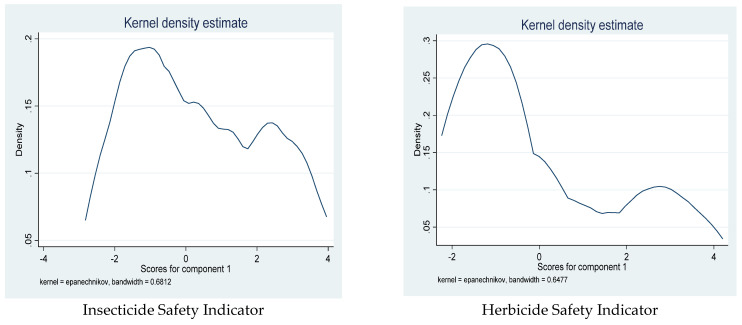

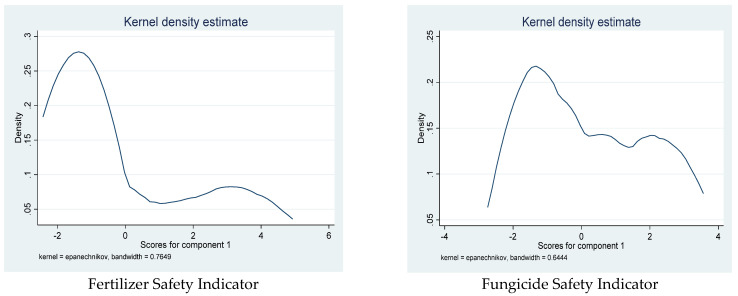
Distributional kernel density graphs of agrochemical safety indicators.

Figure 3 shows the kernel density distributional plots of the agrochemical safety indicators that were generated with the PCA. Moreover, Table 3 shows the descriptive statistics of these safety indicators across the selected LGAs. It reveals that farmers from Yewa South LGA had the highest (0.381) safety indicator for insecticides, while Irewole LGA had the lowest value (−1.196). It also shows that, for herbicide and fertilizer application, the highest average safety indicators were for Ayedaade and Yewa South LGAs, respectively. Yewa South LGA also recorded the highest safety indicator in the handling of fungicides.

### 3.2. Determinants of Agrochemical Safety Compliance Indicators among Cocoa Farmers

The results in Table 4 are for Instrumental Variable Tobit regression analyses. The results show that the Wald Chi Square statistics are all statistically significant (*p* < 0.01). This implies that the model produced good fits for the data and the estimated parameters cannot be said to be jointly equal to zero in each of the results. It also implies that, as hypothesized for this study, we cannot accept the null hypothesis. The Wald’s tests of exogeneity reject the presence of endogeneity. It should be noted that the absence of adequate instruments may have affected the performances of each of the estimated models. The parameters were all estimated with robust standard errors to correct for the presence of heteroscedasticity. In addition, the explanatory variables were examined for the presence of multicollinearity using the variance inflation factor (VIF), which showed a very low value (1.42) that implies the absence of significant multicollinearity.

The results further show that the parameters of the number of PPE owned by the farmers consistently had a positive sign and was statistically significant (*p* < 0.01) in all the estimated models. These results imply that an increase in the number of the PPE owned will result in an increase in the safety compliance indicators. More importantly, the magnitude of the impacts would be highest for insecticides, where the variable has a coefficient of 0.8349. Fungicides and herbicides have the next highest values, with 0.7376 and 0.7318, respectively.

Furthermore, the parameters of marital status and gender did not show statistical significance (*p* > 0.10) in each of the models. Similarly, household size parameters are all with positive signs and statistically insignificant (*p* > 0.10). Farming experience parameters show statistical significance with positive signs in the herbicide (*p* < 0.05) and insecticide (*p* < 0.10) models. These results imply that herbicide and insecticide compliance indicators significantly increased with an increase in farming experience. The parameter of age, in the fertilizer model also, shows statistical significance (*p* < 0.05), with a negative sign. This implies that as farmers’ age increased, compliance with safety guidance in the application of fertilizers decreased. 

The education variable shows statistical significance in the estimated models for fungicides, fertilizers and insecticides. Specifically, the farmers that had no formal education had significantly lower fungicide and insecticide safety compliance indicators. However, the farmers that had no formal education had significantly higher fertilizer safety compliance indicators. In the same vein, the parameters of awareness of manufacturers’ instructions are statistically significant (*p* < 0.01) in all the models, with a positive sign. However, the parameter for herbicides (3.1094) had the highest contribution to the safety compliance indicator. These results generally reveal that cocoa farmers who were aware of agrochemical manufacturers’ instructions had significantly higher safety compliance indicators. Among the health status perception variables, the farmers who perceived health to be good had significantly higher fertilizer safety compliance indicators (*p* < 0.01). However, perception of health status as fair reduced the herbicide safety compliance indicator. Perception of poor health status also significantly increased all the safety compliance indicators (*p* < 0.01), with herbicides having the highest impact (2.9827). 

## 4. Discussion

Cocoa farming across many African countries is dominated by male farmers [39,40]. The findings showed that about nine out of every ten cocoa farmers were males. The underlying tediousness of cocoa agriculture and its labour intensiveness often limit the involvement of women [41]. Even if a woman owns a cocoa farm, the labour input of hired labour may be frequently engaged for activities, such as farm clearing, stumping and application of agrochemicals [42]. More importantly, in many cocoa-growing communities, some women can only assume ownership of cocoa farms by inheritance after the demise of their parents or husbands. In some instances, the traditional land tenure systems may also act as a significant barrier to women’s quest to own a personal cocoa farm [43]. 

The fact that cocoa was the primary occupation of most of the farmers also implies that cocoa land areas must be sufficiently large to cater for their averagely large household sizes. However, with average farm size of less than 8 hectares, the farm sizes were relatively okay, provided they did not comprise more aged cocoa trees. It was noted that 80% to 90% of cocoa output is derived from farms of small sizes, ranging between 2 and 5 hectares [44]. The notion of cocoa land area is of critical importance for whether the farmers are making enough returns to guarantee any cogent farm investments. Therefore, small farm sizes and low cocoa productivity are critically implicated in the low standard of living among many cocoa farmers [45]. Fragmentation of cocoa land holdings due to patriarchal inheritance of cocoa farms is also undermining the financial sustainability of production, with few incentives to procure PPE. The role of sufficient farm income cannot be overemphasized because in some instances, farmers would go for cheaper unapproved agrochemicals due to financial limitations [34].

Further, about one out of every four cocoa farmers never attended school. This is a prevailing trend in Nigerian cocoa agriculture [46]. Similar findings were reported among their counterparts in Indonesia [47], Cote D’Ivoire [48] and Cameroon [30]. Although effective conduct of routinely engaged farming operations on cocoa farms requires no special education, the role of education in fostering awareness of safety requirements in handling productivity cannot be overemphasized [49]. In the absence of other informal training, adequate education is also fundamental in ensuring farmers’ awareness of safety precautions to follow while handling agrochemicals [30]. This assertion can be buttressed from the finding of this study that indicates safety compliance indicators for fungicides and insecticides are significantly lower among illiterate cocoa farmers. In a similar study, in Cameroon, it was found that the attainment of formal education influenced the utilization of PPE among cocoa farmers in Cameroon [30]. The International Labour Organization (ILO) [18] also submitted that the competency that is required for safe handling of agrochemicals cannot be fully achieved without adequate educational levels and targeted training. Education and training can also facilitate farmers’ understanding of the toxicity property in agrochemicals. 

Awareness of the safety precautions to be followed while administering agrochemicals was very high among cocoa farmers. As expected, the parameters of this variable also increased safety compliance indicators across all the estimated models. The majority of the farmers were not eating or drinking during the application of agrochemicals. Given the high toxicity level of agrochemicals, the importance of avoiding food and drink while handling them cannot be overemphasized [30]. This buttresses the results on the high level of awareness on the safety precautions to be followed while handling agrochemicals, which was found to enhance safety compliance. It should also be noted that due to individual differences, some farmers may be aware of safety procedures but refuse to comply. Such risky behaviour is often exhibited out of ignorance and had been reported by Miyittah et al. [28]. Sharifzadeh et al. [50] indicated that farmers may be aware of the associated risks in mishandling agrochemicals but show no compliance towards the enhancement of their safety. 

Ownership of PPE among cocoa farmers was very low. Ntow et al. [51] found that full PPE was only used by 32% of the farmers. Some farmers prefer to own PPE, such as safety boots, with multiple uses rather than investing in those ones that would only find relevance while spraying agrochemicals. This finding is very worrisome given that cocoa agriculture requires significant inputs of agrochemicals. Therefore, the notion of productivity enhancement as a fundamental objective of every farmer requires sufficient utilization of chemical inputs, with fungicide and insecticide application most notable. Possession of PPE is fundamentally necessary to avoid the health hazards that are associated with mishandling and complacency in following manufacturers’ instructions. More importantly, it was found that an increase in the number of PPE owned increased safety compliance indicators across all types of agrochemicals that were studied. This result can be buttressed by the findings of Damalas and Koutroubas [52], who reported that proper usage of PPE should be executed at every stage of agrochemical handling to reduce direct contact, exposure and associated health consequences.

More importantly, some farmers may own PPE and refuse to wear it because of some inconveniences they experience when they are worn. This had been buttressed by the findings of Osei-Owusu and Owusu-Achiaw [12], who reported that 30.50% of Ghanaian cocoa farmers indicated that they were not able to wear masks and goggles due to inconvenience. In a study in Ghana, female farmers were unable to were PPE due to it being inconvenient [28]. The implication of the finding on child’s health was also raised because women who are breastfeeding can pass agrochemical residues to their children through breast milk [53]. This was confirmed by Ntow et al. [54], who reported contamination of female cocoa farmers’ breastmilk in Ghana with traces of organo-chlorine pesticides and dichlorodiphenyltrichloroethane (DDT), which are components of agrochemicals being used on cocoa farms.

An association was also found between perceived health status of cocoa farmers and safety compliance indicators. Specifically, those farmers who indicated their health status as fair had higher safety compliance indicators across all the agrochemicals. This is in accordance with expectation, since the impacts of mishandling of agrochemicals on the health of farmers remains a major public-health concern [18]. The understanding of one’s health status can, therefore, facilitate compliance with safety regulations [27,50,55,56,57,58]. Atu [59] emphasized the toxicity of agrochemicals with significant implications on human health. More importantly, WHO/UNEP [60] reported misuse of pesticides causes about 3 million emergency poisoning cases among farm workers and about 20,000 deaths largely in developing countries. In some previous studies, pesticide operators complained of stomach poisoning, skin and eye irritations [27,55,61].

The results also showed some positive impacts of farming experience in promoting safety compliance indicators for herbicides and insecticides. This is expected because well-experienced cocoa farmers would understand the risks that are associated with the mishandling and misuse of agrochemicals. Experience can also be fundamental in the awareness of routine precautions to be carried out while spraying agrochemicals. In a similar study, Miyittah et al. [28] found that years of cocoa farming did not translate into wearing of PPE during agrochemical application. 

## 5. Conclusions

This study analysed the factors influencing compliance with agrochemical safety. The findings revealed the need for significant informal training for the farmers, especially for those without formal education, on the safe utilization of agrochemicals to minimize the associated health risks. Such training would facilitate awareness of safety precautions that are often prescribed by agrochemical manufacturers and they can be in the form of Farmers’ Field School (FFS) and media programmes on some local radio stations that are easily accessible to cocoa farmers. Such training should generally raise awareness on manufacturers’ instructions on different forms of agrochemicals, to facilitate compliance with safety requirements. It was found that farmers that rated personal health as fair had higher compliance with agrochemical safety precautions. There is, therefore, a need to ensure adequate knowledge of the health risks that are associated with the misuse of agrochemicals among cocoa farmers. Cocoa farmers need to understand the cumulative health impacts of exposure to agrochemicals over time and that safety consciousness should not be the prerogative only when they are sick.

## Figures and Tables

**Table 1:** Distribution of sampled cocoa farmers across the villages.

Osun State				Ogun State			
Ayedaade		Irewole LGA		Yewa North		Yewa South	
Location	Frequency	Location	Frequency	Location	Frequency	Location	Frequency
Aba Otun	7	Agbopo	7	Igbogila	3	Eredo	4
Ago Igbira	3	Baraloje	7	Igbota	3	Idogo	4
Agodo	1	Fagbemi	2	Ijowun	3	Igbo Igbin	45
Akinrepo	8	Odeyinka	16	Imasayi	1	Ijado	1
Akorede	1	-	-	Owode Ketu	25	Ilaro	2
Bembe	11	-	-	Sawanjo	85	Itoro	8
Mokore I, II, III	19	-	-	Tata	36	Iweke	2
-	-	-	-	-	-	Odan-Itoro	2
-	-	-	-	-	-	Olokuta	18
-	-	-	-	-	-	Orile-Itoro	2
Total	50	-	32	-	156	-	88

**Table 2:** Descriptive statistics of cocoa farmers’ selected demographic characteristics.

	Yewa North (*n* = 156)		Yewa South (*n* = 88)		Ayedaade (*n* = 50)		Irewole (*n* = 32)		All Farmers (*n* = 326)	
	Mean	Std Dev	Mean	Std Dev	Mean	Std Dev	Mean	Std Dev	Mean	Std Dev
Married	0.019		0.091		0.060		0.063		0.049	
Male	0.936		0.886		0.880		0.969		0.917	
No Education	0.231		0.409		0.260		0.156		0.276	
Primary Occupation (Farming)	0.904		0.864		0.940		0.875		0.896	
Household Size	7.064	3.592	7.773	4.784	7.440	4.427	7.781	4.757	7.383	4.183
Cocoa Farming Experience	23.936	16.188	22.091	16.018	23.460	15.695	22.750	14.560	23.248	15.864
Age of Farmers	50.032	16.181	52.705	15.690	51.960	16.411	51.563	15.604	51.199	15.999
Number of PPE owned	1.2179	1.6629	2.2955	1.8579	2.1400	1.8953	0.4375	0.8776	1.574	1.799
Cocoa land areas	6.3590	6.8866	8.1193	9.3931	9.1000	9.8768	9.0000	11.3216	7.514	8.632
Perceived Health as Excellent	0.288		0.261		0.160		0.313		0.264	
Perceived Health as Very Good	0.442		0.523		0.420		0.344		0.451	
Perceived Health as Good	0.237		0.182		0.300		0.281		0.236	
Perceived Health as Fair	0.026		0.034		0.120		0.063		0.046	
Perceived Health as Poor	0.006		0.000		0.000		0.000		0.003	

Cocoa farmers’ awareness of manufacturers’ instructions and safety compliance.

**Table 3:** Descriptive statistics of cocoa farmer indicators of agrochemical safety compliance.

Compliance Index	Yewa North (*n* = 136)		Yewa South (*n* = 88)		Ayedaade (*n* = 50)		Irewole (*n* = 32)		All Farmers (*n* = 326)	
	Mean	Std Dev	Mean	Std Dev	Mean	Std Dev	Mean	Std Dev	Mean	Std Dev
Insecticides	−0.491	1.543	0.381	1.853	0.089	1.998	−1.196	0.871	−0.236	1.724
Herbicides	−0.328	1.367	−0.079	1.762	−0.012	1.748	−1.103	0.833	−0.288	1.529
Fertilizers	−0.366	1.617	−0.078	2.081	−0.185	2.114	−1.273	0.698	−0.350	1.799
Fungicides	−0.509	1.440	0.230	1.753	0.131	1.644	−1.069	0.794	−0.266	1.569

**Table 4:** Results of instrumental variable Tobit regression of the determinants of safety compliance.

	Fungicides		Fertilizers		Herbicides		Insecticides	
Variables	Coefficients	Robust Std Error	Coefficients	Robust Std. Error	Coefficients	Robust Std. Error	Coefficients	Robust Std. Error
PPE Owned	0.7376 ***	0.0700	0.4962 ***	0.0917	0.7318 ***	0.1247	0.8349 ***	0.0590
Married	0.0329	0.1434	0.0218	0.2598	−0.1642	0.2819	−0.0227	0.1539
Male	−0.0637	0.1322	−0.1366	0.2030	−0.3585	0.2838	−0.1503	0.1756
No Education	−0.2131 **	0.1045	0.2257 *	0.1230	−0.1653	0.1364	−0.1327 *	0.0788
Primary Occupation (Farming)	0.1367	0.1501	0.1182	0.2185	0.0485	0.1946	0.0584	0.1417
Household Size	0.0132	0.0080	0.0101	0.0142	−0.0035	0.0165	0.0084	0.0098
Cocoa Farming Experience	0.0024	0.0029	0.0038	0.0046	0.0100 **	0.0049	0.0042 *	0.0024
Age of Farmers	−0.0003	0.0037	−0.0115 **	0.0050	−0.0064	0.0061	0.0001	0.0031
Aware of Manufacturers’ Instructions	1.3051 ***	0.2389	1.9615 ***	0.1144	3.1094 ***	0.2186	1.2036 ***	0.2838
Perceived Health as Very Good	−0.0156	0.1106	0.2178	0.1424	−0.1850	0.1752	0.0329	0.0986
Perceived Health as Good	−0.0488	0.1207	0.4022 ***	0.1552	−0.0450	0.2094	−0.0146	0.1137
Perceived Health as Fair	0.0419	0.1726	0.2120	0.2468	−0.4537 ***	0.2067	−0.0933	0.2162
Perceived Health as Poor	1.1813 ***	0.2354	1.7144 ***	0.1731	2.9827 ***	0.3078	1.1113 ***	0.2791
Constant	−2.7315 ***	0.2876	−2.0221 ***	0.3996	−3.2393 ***	0.5056	−2.6944 ***	0.3224
corr(e.ppeowned,e.insectindica)	0.0256	0.1851	0.1050	0.1546	−0.2269	0.2093	0.0999	0.1572
sd(e.insectindica)	0.6860	0.0716	1.0148	0.0450	0.9847	0.0855	0.6102	0.0656
sd(e.ppeowned)	1.5930	0.0557	1.6127	0.0586	1.6307	0.0577	1.5988	0.0555
Number of observations	326		326		326		326	
Log pseudolikelihood	−942.94		−1083.93		−948.61		−911.77	
Wald chi2(12)	2449.03 ***		473.78 ***		1148.62 ***		4182.60 ***	
Wald test of exogeneity	0.02		0.45		1.10		0.40	

Note: ***—Significant at 1% level; **—Significant at 5% level; *—Significant at 10% level.

## Data Availability

Data for this study can be made available upon reasonable request.
